# Predictors of persistent fatigue one year after severe COVID-19: A prospective cohort study on depression and lung function

**DOI:** 10.1016/j.clinsp.2025.100846

**Published:** 2025-11-19

**Authors:** Elizabeth Mendes da Silva, Camila Machado de Campos, Caroline Gil de Godoy, Danielle Brancolini de Oliveira, Angelica Castilho Alonso, Vanderlei Carneiro da Silva, Ana Carolina Basso Schmitt, Gabriela Sayuri Ochiai, Bianca de Oliveira Candido Viana, Juliana Magalhães da Silva, Celso Ricardo Fernandes de Carvalho, Carlos Roberto Ribeiro de Carvalho, Julia Maria D'Andréa Greve, José Eduardo Pompeu

**Affiliations:** a Department of Physical Therapy, Speech and Occupational Therapy, Faculdade de Medicina, Universidade de São Paulo, São Paulo, SP, Brazil; b Movement Studies Laboratory, Instituto de Traumatologia e Ortopedia (IOT), Hospital das Clínicas, Universidade de São Paulo, São Paulo, SP, Brazil; c Pneumology Division, Cardiopulmonary Department, Instituto do Coração do Hospital das Clínicas, Universidade de São Paulo, São Paulo, SP, Brazil

**Keywords:** Post-acute covid-19 syndrome, Fatigue, Hospitalization, Depression, Lung capacity, Prospective Cohort Study

## Abstract

**Background:**

Fatigue is among the most enduring symptoms of long COVID. However, the relationship between persistent fatigue and factors such as physical performance, lung function, anxiety, and depression in severe cases of COVID-19 remains underexplored.

**Objectives:**

To assess the prevalence of fatigue and its association with these factors in patients one year after severe COVID-19 hospital discharge.

**Methods:**

This cohort comprised participants aged 18-years and older diagnosed with COVID-19. Evaluations were conducted at one, four, six-, and twelve-months following hospital discharge. Clinical data were collected, which included assessments of fatigue – Functional Assessment of Chronic Illness Therapy Fatigue subscale, lung capacity ‒ Spirometry, physical performance – 1-minute Sit to Stand Test, psychological aspects – Hospital Anxiety and Depression Scale, and functional capacity *–* Barthel index.

**Results:**

Of the 162 participants, 50 % experienced fatigue one-month post-discharge and this prevalence gradually decreased to 36 % by the end of one-year period. The fatigue group had a median age of 58-years. It was predominantly composed of women, with a majority identifying as non-white and having a body mass index greater than 30 kg/m^2^. Physical performance assessments within the fatigue group showed no significant changes over time, whereas lung capacity demonstrated significant improvement only at the 12-month mark. Additionally, anxiety and depression levels were significantly higher in the fatigue group over time (*p* < 0.05).

**Conclusions:**

Fatigue persisted in 36 % of participants one-year post-hospital discharge. The potential predictors of long-term fatigue were depressive symptoms and impaired lung function observed within the first month after hospital discharge. Therefore, the authors highlight the importance of screening and monitoring these aspects in order to enable early intervention in this population.

## Highlights

• Fatigue is a persistent symptom that often resists complete recovery in many cases.
• Patients experiencing fatigue exhibited minimal variation in functional capacity after 12 months.
• Anxiety, depression, and pulmonary impairments are associated with long-term fatigue.
• Comprehensive screening for post-COVID-19 fatigue is essential for effective management.

## Introduction

Following the rapid spread of COVID-19, which resulted in approximately 772 million confirmed cases and 6.9 million deaths worldwide, Brazil reported around 37.5 million confirmed cases and approximately 714 thousand deaths. The World Health Organization (WHO) lifted the pandemic on May 5, 2023.^1^ Nevertheless, COVID-19 continues to pose a significant global public health challenge, as a substantial number of individuals are left recovering from both physical and mental sequelae. Notably, about 30 % of those infected required hospitalization,^2^ prompting the scientific community to seek a deeper understanding of the long-term impacts on survivors.

Among the symptoms associated with long COVID ‒ defined as the prolonged persistence of symptoms ‒ fatigue has been identified as one of the most prevalent and debilitating complaints reported by patients.^3^, ^4^, ^5^, ^6^, ^7^ Fatigue is characterized by low energy, extreme tiredness, muscle weakness, pain, difficulty concentrating, and changes in sleep and mood, all of which can severely compromise quality of life and hinder daily functioning.^8^ Additionally, these manifestations may have significant economic implications.

The lungs are recognized as the organs most affected by COVID-19. When the virus infects the respiratory system, pulmonary infiltrates obstruct gas exchange, resulting in inadequate oxygen supply for mitochondrial bioenergetics.^9^ This compromise, alongside physical deconditioning due to hospitalization, may exacerbate muscle damage ‒ particularly in the diaphragm and lower limb muscles.^10^ The susceptibility of skeletal muscles to the SARS-CoV-2 virus appears to stem from the direct infection of cells abundant in ACE-2 receptors and indirectly from systemic cytokine release, which disrupts homeostasis and accelerates muscle loss.^11^^,^^12^ Furthermore, the virus may directly invade the Central Nervous System (CNS) via the olfactory bulb ‒ a dopamine-rich area linked to motivation, pleasure, and action ‒ resulting in altered dopamine levels and other neurotransmitters. Indirectly, the combination of systemic inflammatory responses, such as the “cytokine storm” and hypoxia resulting from respiratory failure, may exacerbate psychological symptoms or worsen pre-existing disorders during both the acute and chronic phases of COVID-19.^13^^,^^14^

Given that fatigue is a multifactorial symptom influenced by neurotransmitter imbalances, inflammatory mechanisms, and psychological disorders, its etiology and impact in the context of post-COVID-19 remain uncertain due to the complexity of the interacting systems involved.^15^ While prior studies have explored persistent symptoms of long COVID, there is still a lack of data regarding the persistence of fatigue among COVID-19 survivors. This cohort study investigated the clinical presentation of fatigue and its association with physical performance, lung capacity, and psychological well-being. Furthermore, the study aimed to identify factors related to fatigue over one year among patients hospitalized due to COVID-19 at a tertiary hospital in São Paulo, Brazil.

## Materials and methods

This prospective cohort study included patients aged 18-years and older, both sexes, who were hospitalized for over 24-hours following a confirmed diagnosis of SARS-CoV-2 through RT-PCR (Reverse Transcription Polymerase Chain Reaction) and traditional serology tests at the Hospital das Clínicas of the Faculty of Medicine of the University of São Paulo (HCFMUSP), a recognized reference hospital in São Paulo, Brazil. This study is part of the project entitled “Functional prognosis of adults and elderly people after hospitalization due to COVID-19″,^16^ approved by the Hospital das Clínicas Ethics Committee, with the approval number 4.052.246.

All eligible participants were contacted by telephone and invited to appear in person at the collection site on the day of the first assessment. Only those who, after reading the full Informed Consent Form (ICF), expressed their agreement by signing it were included in the study. Financial assistance was offered to cover the costs of transportation and meals. Participants had to be hemodynamically stable during evaluations, possess preserved or corrected visual and auditory acuity, and demonstrate the ability to understand simple commands.

A convenience sampling method was employed, with participants undergoing an evaluation protocol at four time points: 1-month, 4-months, 6-months, and 12-months post-hospital discharge, from June 2020 to July 2022.

To minimize the potential for attrition bias, the authors implemented strategies throughout the study to facilitate effective communication with participants ‒ including telephone contact for pre-appointment confirmation ‒ and actively promoted engagement to reduce the risk of loss to follow-up.

Data collection encompassed sociodemographic information such as age, gender, Body Mass Index (BMI), education level, marital status, hospitalization history, and clinical characteristics from all participants (Table 1).

**Table 1:** General characteristics of the participants after 1-, 4-, 6- and 12-months after hospital discharge due to COVID-19.

	1-month, *n* = 162		4-month, *n* = 100		6-month, *n* = 87		12-month, *n* = 88	
	Fatigue < 43pts	Non-fatigue > 43pts	Fatigue < 43pts	Non-fatigue > 43pts	Fatigue < 43pts	Non-fatigue > 43pts	Fatigue < 43pts	Non-fatigue > 43pts
**Sociodemographic characteristics**								
**Age (years)**	*n* = 162 (81/81)		*n* = 100 (49/51)		*n* = 87 (36/51)		*n* = 88 (32/56)	
	58 (48‒65)	59 (52‒67)	62 (49‒69)	57 (48.5‒63)	59.5 (50‒71)	59 (50‒67)	58 (47.5‒65)	58 (49‒65)
**Sex**	*n* = 162 (81/81)		*n* = 100 (49/51)		*n* = 87 (36/51)		*n* = 88 (32/56)	
Male	35 (43 %)	54 (67 %)	22 (45 %)	32 (63 %)	15 (42 %)	34 (67 %)	14 (44 %)	36 (64 %)
Female	46 (57 %)^a^	27 (33 %)	27 (55 %)	19 (37 %)	21 (58 %)^a^	17 (33 %)	18 (56 %)	20 (36 %)
**BMI (kg/m^2^)**	*n* = 160 (79/81)		*n* = 99 (48/51)		*n* = 86 (35/51)		*n* = 87 (32/55)	
	30 (27‒33.4)	28.5 (27‒31.9)	30 (27‒33)	30 (27‒33)	30 (27‒33)	28 (26‒32)	30,5 (28‒33)	28 (26‒31.5)
**Ethnic group**	*n* = 161 (81/80)		*n* = 98 (48/50)		*n* = 86 (35/51)		*n* = 87 (32/55)	
White	33 (41 %)	47 (59 %)	20 (42 %)	22 (44 %)	16 (46 %)	24 (47 %)	13 (41 %)	27 (49 %)
Non-white	48 (59 %)	33 (41 %)	28 (58 %)	28 (56 %)	19 (54 %)	27 (53 %)	19 (59 %)	28 (51 %)
**Education**	*n* = 160 (80/80)		*n* = 99 (48/51)		*n* = 86 (35/51)		*n* = 87 (32/55)	
< 8-years	19 (24 %)	24 (30 %)	13 (27 %)	14 (27 %)	11 (31 %)	11 (22 %)	9 (28 %)	15 (27 %)
8‒11 years	48 (60 %)	39 (49 %)	27 (56 %)	28 (55 %)	17 (49 %)	29 (57 %)	17 (53 %)	28 (51 %)
> 11-years	13 (16 %)	17 (21 %)	8 (17 %)	9 (18 %)	7 (20 %)	11 (21 %)	6 (19 %)	12 (22 %)
**Marital status**	*n* = 162 (81/81)		*n* = 100 (49/51)		*n* = 87 (36/51)		*n* = 88 (32/56)	
Single	14 (17 %)	12 (15 %)	9 (18 %)	10 (19 %)	6 (17 %)	6 (12 %)	5 (16 %)	11 (20 %)
Married/Stable union	52 (64 %)	51 (63 %)	30 (61 %)	30 (59 %)	20 (55 %)	33 (65 %)	20 (62 %)	34 (61 %)
Separated/ Divorced	7 (9 %)	11 (14 %)	6 (12 %)	5 (10 %)	5 (14 %)	7 (14 %)	2 (6 %)	8 (14 %)
Widower	8 (10 %)	7 (8 %)	4 (9 %)	6 (12 %)	5 (14 %)	5 (9 %)	5 (16 %)	3 (5 %)
**Characteristics of hospitalization**								
**Length of stay (days)**	*n* = 162 (81/81)		*n* = 99 (48/51)		*n* = 86 (35/51)		*n* = 87 (32/55)	
	22 (15‒36)	21 (13‒30)	25.5 (17‒40)	25 (17‒32)	28 (18‒38)	25 (18‒31)	29.5 (17‒39)	25 (19‒35)
**ICU**	*n* = 161 (80/81)		*n* = 99 (48/51)		*n* = 86 (35/51)		*n* = 87 (32/55)	
No	15 (19 %)	9 (11 %)	8 (17 %)	4 (8 %)	4 (11 %)	5 (10 %)	4 (13 %)	4 (7 %)
Yes	65 (81 %)	72 (89 %)	40 (83 %)	47 (92 %)	31 (89 %)	46 (90 %)	28 (87 %)	51 (93 %)
**ICU time (days)**	*n* = 147 (72/75)		*n* = 90 (43/47)		*n* = 78 (34/44)		*n* = 80 (29/51)	
	9 (2‒16)	9 (5‒18)	9 (2‒17)	12 (6‒21)	13 (7‒22)	10 (6‒14)	14 (8‒18)	9 (5‒17)
**IMV**	*n* = 158 (77/81)		*n* = 97 (46/51)		*n* = 85 (34/51)		*n* = 86 (32/54)	
Não	30 (39 %)	36 (44 %)	19 (41 %)	17 (33 %)	7 (21 %)	16 (31 %)	7 (22 %)	16 (30 %)
Sim	47 (61 %)	45 (56 %)	27 (59 %)	34 (67 %)	27 (79 %)	35 (69 %)	25 (78 %)	38 (70 %)
**Clinical features**								
**FACIT-F**	*n* = 162		*n* = 100		*n* = 87		*n* = 88	
Total score	33 (25‒39)^b^	47 (45‒49)	33 (24‒38)^b^	48 (46‒50)	33 (25‒39)^b^	49 (46‒50)	30 (24‒37)^b^	48 (46‒50)
Fatigue < 43-ponts	81 (50 %)	81 (50 %)	49 (49 %)	51 (51 %)	36 (41 %)	51 (59 %)	32 (36 %)	56 (64 %)
Fatigue < 30-ponts	32 (20 %)	130 (80 %)	16 (16 %)	84 (84 %)	13 (15 %)	74 (85 %)	15 (17 %)	73 (83 %)
**CCI**	*n* = 161 (81/80)		*n* = 99 (49/50)		*n* = 86 (36/50)		*n* = 87 (31/56)	
Total score	3 (2‒5)	3 (2‒5)	3 (2‒5)	3 (2‒4)	3 (2‒6)	3 (2‒5)	3 (2‒6)	3 (2‒4)
**HADS – Depression**	*n* = 161 (81/80)		*n* = 100 (49/51)		*n* = 87 (36/51)		*n* = 88 (32/56)	
Total score	7 (3‒11)^b^	1 (0‒3)	9 (5‒14)^b^	1 (0‒3)	8 (5‒13) b	1 (0‒3)	10 (5‒11)^b^	1 (0‒2)
**Classification**								
No depression	43 (54 %)^b^	76 (95 %)	19 (39 %) b	50 (98 %)	15 (42 %) b	49 (96 %)	11 (34 %)^b^	53 (95 %)
Possible	19 (23 %)	3 (4 %)	12 (24 %)	1 (2 %)	9 (25 %)	2 (4 %)	13 (41 %)	3 (5 %)
Probable	19 (23 %)	1 (1 %)	18 (37 %)	0 (0 %)	12 (33 %)	0 (0 %)	8 (25 %)	0 (0 %)
**HADS – Anxiety**	*n* = 161 (81/80)		*n* = 100 (49/51)		*n* = 87 (36/51)		*n* = 88 (32/56)	
Total score	7 (5‒10)^b^	2 (1‒4)	8 (5‒11) b	2 (1‒4)	8 (5‒12)^b^	2 (1‒4)	8 (6‒12)^b^	2 (1‒4)
**Classification**								
No anxiety	42 (52 %)^b^	72 (90 %)	24 (49 %)^b^	49 (96 %)	16 (44 %)^b^	45 (88 %)	14 (44 %)^b^	53 (94 %)
Possible	23 (28 %)	6 (7 %)	13 (27 %)	1 (2 %)	11 (31 %)	4 (8 %)	10 (31 %)	2 (4 %)
Probable	16 (20 %)	2 (3 %)	12 (24 %)	1 (2 %)	9 (25 %)	2 (4 %)	8 (25 %)	1 (2 %)
**BI**	*n* = 151 (74/77)		*n* = 100 (49/51)		*n* = 86 (36/50)		*n* = 88 (32/56)	
Total score	95 (90‒100)^a^	100 (95‒100)	95 (95‒100)^b^	100 (100‒100)	95 (90‒100)^b^	100 (100‒100)	95 (90‒100)^b^	100 (100‒100)
**1min-STS**	*n* = 139 (66/73)		*n* = 72 (37/35)		*n* = 73 (39/34)		*n* = 68 (24/44)	
Repetitions	15 (11‒20)^a^	19 (14‒22)	17 (14‒20)^a^	19 (17‒22)	17 (13‒21)	21 (18‒23)	16 (12‒21)^a^	21 (18‒23)
**Spirometry**	*n* = 134 (64/70)		*n* = 82 (43/39)		*n* = 63 (27/36)		*n* = 70 (25/45)	
FVC predicted ( %)	73 (65‒81)	77 (66‒86)	77 (70‒88)	79 (71‒84)	78 (70‒85)	82 (72‒89)	81 (61‒91)^a^	86 (80‒94)
FEF predicted ( %)	116 (82‒141)	104 (75‒132)	113 (85‒148)	118 (87‒136)	99 (70‒138)	117 (87‒143	109 (83‒122)	122 (87‒144)
FEV1 predicted ( %)	77 (69‒90)	80 (66‒93)	84 (71‒94)	82 (76‒91)	81 (66‒90)	84 (73‒94)	84 (66‒97)	87 (80‒95)
FEV1/FVC predicted ( %)	110 (104‒115)	106 (101‒113)	108 (102‒112)	109 (102‒113)	108 (98‒109)	106,5 (99‒111)	106 (100‒110)	105 (100‒109)

1 min-STS, 1-min sit-to-stand test; BI, Barthel Index; BMI, Body Mass Index; CCI, Charlson Comorbidity Index; COVID-19, Coronavirus Disease of 2019; FACIT-F, Functional Assessment of Chronic illness therapy-fatigue; FEV1, forced expiratory volume in one second; FEF25−75, forced expiratory flow between 25−75 % of FVC; FVC, Forced Vital Capacity; HADS, Hospital Anxiety and Depression; ICU, Intensive Care Unit; IMV, Intubation and Mechanical Ventilation.

Data are shown as median (IQR) or n ( %). The table summarizes the differences between the groups regarding sociodemographic, hospital and clinical characteristics in the four assessments.

^ab^Significant difference between fatigue and non-fatigue groups (*p* < 0.05 and *p* < 0.0001, respectively).

### Fatigue

Fatigue was evaluated using the Functional Assessment of Chronic Illness Therapy Fatigue subscale (FACIT-F), initially developed to identify and assess fatigue in cancer patients and subsequently validated for various clinical conditions.^17^ The FACIT-F consists of 13-items, with responses ranging from zero to four points, corresponding to “none” to “very much”, respectively. The total possible score is 52-points, with higher scores indicating a lower perception of fatigue. Due to the absence of cut-off values for the Brazilian population, the authors adopted the American population's established threshold, wherein fatigue is defined as a score below 43-points.^18^

### Lung capacity

The authors assessed lung capacity via pulmonary function tests (spirometry), which measure the volume of air inhaled and exhaled and respiratory flow rates.^19^ Evaluations were conducted using a portable digital spirometer (Microquark Cosmed, Italy), adhering to the guidelines established by the American Thoracic Society and the European Respiratory Society.^20^ The following variables were recorded as absolute and predicted values: Forced Vital Capacity (FVC), Forced Expiratory Volume in one second (FEV1), FEV1/FVC ratio, and forced expiratory flow (FEF_25‒75__%_).

### Anxiety and depression

Symptoms of anxiety and depression were assessed using the Hospital Anxiety and Depression Scale (HADS).^21^ This tool comprises 14-multiple-choice questions, divided into two independent scales with seven items each ‒ one assessing Anxiety (HADS-A) and the other evaluating Depression (HADS-D). Each subscale yields a total score ranging from 0 to 21-points, interpreted as follows: 0–7 = Unlikely; 8–10 = Possible; and 11–21 = Probable.^22^

### Physical performance

Physical performance was measured using the 1-minute Sit-to-Stand Test.^23^ During the assessment, participants were instructed to sit and stand from a standard-height chair (46 cm) without support, with their knees and hips bent at 90 degrees and feet slightly apart on the floor. The authors recorded the number of repetitions completed within one minute.

### Statistical analysis

A descriptive analysis was conducted to summarize participant characteristics across evaluations, with continuous data presented as medians and interquartile ranges (IQR_25‒75__%_) and categorical data expressed as absolute values (n) and percentages ( %).

Fatigue was identified based on the FACIT-F score, allowing the classification of participants into fatigue and no fatigue groups. The non-parametric Mann-Whitney *U* test was used to compare medians between groups, while the Chi-Square test was used for categorical data comparisons. Considering the reduction in sample size over the follow-up period, the authors adopted Generalized Linear Mixed Models (GLMM) to analyze the longitudinal data, which are robust to the presence of missing data under the Missing-At-Random (MAR) assumption. This approach allows the use of all available observations, maintaining statistical power and avoiding the bias associated with excluding participants with incomplete data.^24^^,^^25^ Thus, even with the decrease in the sample size over time, the estimates remain reliable, as recommended in the specialized literature.^24^^,^^25^ Therefore, an analysis was performed using a Generalized Linear Mixed Model (GLMM), with group and time considered as fixed effects, and time treated as a repeated measure. The interaction between group and time was also included as a fixed effect. For multiple comparisons, the Bonferroni post hoc test was applied.

Furthermore, the decision tree model^26^ was trained to identify the main factors associated with long-term fatigue, using an algorithm that iteratively evaluates splits in the data based on information gain. This type of model is particularly robust when dealing with multiple predictors. The algorithm automatically explores combinations between variables, such as BMI and depressive symptoms, and selects the most informative cutoff points to classify participants. The model's performance was assessed by overall accuracy, and the identified predictors reflect those with the greatest discriminative power in the analyzed sample.

The study's data analyses were conducted using SAS v.9.4 for descriptive statistics and *R* statistical package version 4.2.2 for inferential statistics, with a significance level set at *p* < 0.05.

## Results

**Fig. 1:**
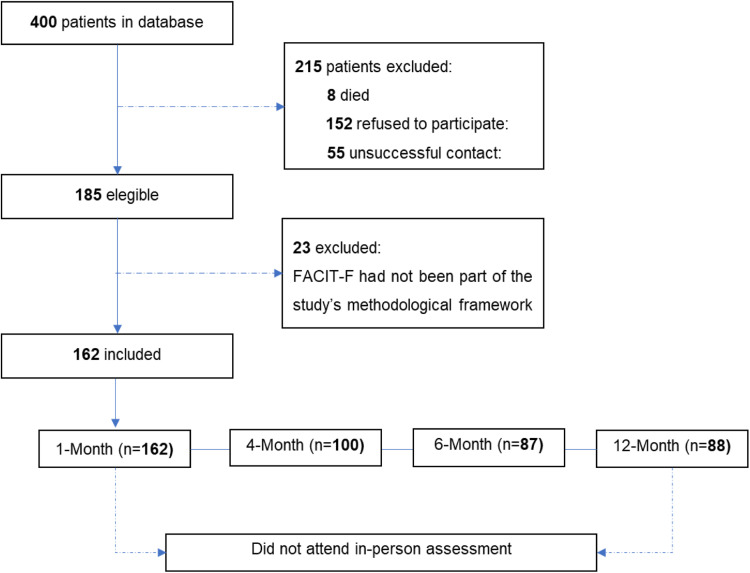
Flowchart of participants.

A total of 185 participants were initially selected for the study; however, 23 participants (12.4 %) did not complete the fatigue questionnaire during the first assessment and were consequently excluded (Fig. 1).

The flowchart of the study population in this cohort.

As a result, 162 participants were included in the analysis. Table 1 presents the main characteristics of the participants, alongside the prevalence of fatigue symptoms assessed at 1-, 4-, 6-, and 12-months following hospital discharge. The four evaluations noted that the fatigue group predominantly comprised females, with a median age ranging from 58 to 62 years (IQR 48‒71). The group was characterized by a median Body Mass Index (BMI) of 30 kg/m^2^ (IQR 26.0‒33.4) and a majority identifying as non-white. Regarding hospitalization data, in this group, 81 % (*n* = 65/80) were admitted to the Intensive Care Unit (ICU), and 61 % (*n* = 47/77) required invasive mechanical ventilation (Table 1).

### Fatigue

The presence of fatigue symptoms was identified in 50 % (*n* = 81/162) of participants one month after hospital discharge, followed by 49 % (*n* = 49/100) at four months, 41 % (*n* = 36/87) at 6-months, and 36 % (*n* = 32/88) at twelve months, indicating a gradual decrease in prevalence over the year (Table 1). Comparisons between groups revealed significant differences in FACIT-F scores, with the fatigue group scoring lower than the non-fatigue group at all assessment time points (*p* < 0.001).

### Physical performance

Participants in the fatigue group performed fewer repetitions in the 1-Minute Sit-to-Stand Test (1m-STS), demonstrating lower physical performance compared to those without fatigue. Significant differences were observed at one month (*p* = 0.0045), four months (*p* = 0.01), and twelve months (*p* = 0.001), although the difference at six months was not statistically significant (*p* = 0.06). Over the 12-month period, there was no significant change in physical performance within the fatigue group, suggesting that performance in the 1m-STS remained stable compared to the initial assessment (Table 2).

**Table 2:** Analysis of the interaction between the fatigue group and the other independent variables over time.

	Interaction: Fatigue Group * Independent variables * Time								
Variables	1 – 4 month			1 – 6 month			1 – 12 month		
Lung capacity	Estimate	Standard error	p	Estimate	Standard error	p	Estimate	Standard error	p
FVC predicted ( %)	−2.749	1.889	0.147	−4.871	2.101	0.021^a^	−4.343	2.059	0.036^a^
FEF predicted ( %)	1.342	7.6245	0.860	4.2519	8.2971	0.609	4.7806	8.1269	0.557
FEV1 predicted ( %)	0.3094	5.2882	0.9534	−1.1479	5.8773	0.8453	−8.6571	5.7513	01,337
FEV1/FVC predicted ( %)	0.542	1.3771	0.694	0.411	1.5054	0.785	1.3577	1.4752	0.358
Physical performance									
1 min-STS	−0.569	0.977	0.561	1.1302	1.0319	0.275	−0.990	1.0257	0.336
Psychological aspects									
HADS – Anxiety	−1.0292	0.811	0.205	−2.2486	0.820	0.007^a^	−2.2707	0.830	0.007^a^
HADS – Depression	−1.0525	0.794	0.186	−2.4005	0.804	0.003^a^	−2.5112	0.813	0.002^a^
Functional capacity									
BI	1.6801	1.7557	0.339	2.0067	1.8448	0.278	0.028	1.8346	0.988

1min-STS, 1-min Sit-to-Stand Test; FACIT-F, Functional Assessment of Chronic Illness Therapy-Fatigue; FEV1, Forced Expiratory Volume in one second; FEF_25−75__%_, Forced Expiratory Flow between 25−75 % of FVC; FVC, Forced Vital Capacity; HADS, Hospital Anxiety and Depression.

Interaction between the presence of fatigue symptoms and chance in outcomes over the 12-months after hospital discharge.

a Significant difference (*p* < 0.05).

### Lung capacity

In the fatigue group, there was a gradual increase in predicted values for Forced Vital Capacity (FVC) between the first and sixth months (*p* = 0.02) and between the first month and twelve months (*p* = 0.03). As described in Table 1, the non-fatigue group achieved FVC values within the normal range (above 80 % of the predicted value) starting at six months. In contrast, the fatigue group experienced significant improvement in FVC only by twelve months post-discharge, with this difference between groups becoming significant at the final evaluation (*p* = 0.04). Other pulmonary function test variables, including Forced Expiratory Flow (FEF), Forced Expiratory Volume in one second (FEV1), and the FEV1/FVC ratio, did not show statistically significant changes over the 12-months (*p* > 0.05; Table 2).

### Psychological aspects

Of the 162 participants evaluated, only one did not complete the fatigue screening questionnaire during the first assessment and was excluded.

#### Anxiety

**Fig. 2:**
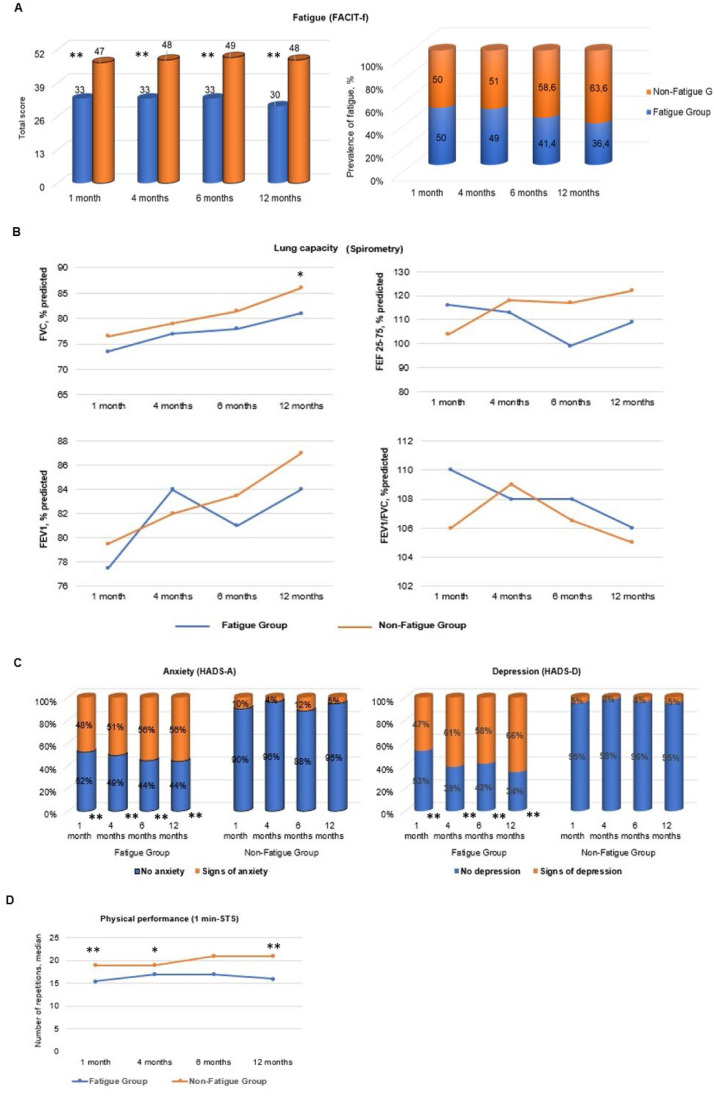
Characterization of the long-term post-COVID-19 fatigue symptom and the clinical outcomes. 1min-STS, 1-min Sit-to-Stand Test; COVID-19, Coronavirus Disease of 2019; FACIT-F, Functional Assessment of Chronic Illness Therapy-Fatigue; FEV1, Forced Expiratory Volume in one second; FEF_25−75__%_, Forced Expiratory Flow between 25−75 % of FVC; FVC, Forced Vital Capacity; HADS, Hospital Anxiety and Depression.

Data indicated that 48 % (*n* = 39/81) of participants in the fatigue group exhibited possible or probable anxiety one-month post-discharge, increasing to 51 % (*n* = 25/49) at four months, 56 % (*n* = 20/36) at six months, and remaining at 56 % (*n* = 18/32) at twelve months. Significant differences existed between the groups at all four time points (*p* = 0.0001). Additionally, there was a trend toward increasing HADS-A scores in the fatigue group between the first and sixth months and again between the first and twelfth months (*p* = 0.007 in both instances), suggesting worsening anxiety symptoms over time (Fig. 2).

#### Depression

Depressive symptoms in the fatigue group were reported by 47 % (*n* = 38/81) of participants in the first evaluation, followed by 61 % (*n* = 30/49), 58 % (*n* = 21/36), and 66 % (*n* = 21/32) at subsequent assessments (Table 1). The two groups differed significantly at all evaluation points (*p* = 0.0000), with signs of depression noted in the fatigue group shortly after the fourth month post-discharge (Fig. 2). Additionally, a significant time effect was observed, indicating a deterioration in depressive symptoms among fatigued participants between the first and sixth months (*p* = 0.031) and between the first and twelfth months (*p* = 0.0022; Table 2).

This figure presents (A) the prevalence of fatigue symptoms. Panels (B), (C), and (D) illustrate the evolution of lung capacity, psychological aspects (anxiety and depression), and physical performance, comparing the fatigue and non-fatigue groups over the 12-month follow-up period after hospital discharge due to COVID-19. ^*,**^Significant difference between fatigue and non-fatigue groups (*p* < 0.05 and *p* < 0.0001, respectively).

#### Predictors of fatigue

**Fig. 3:**
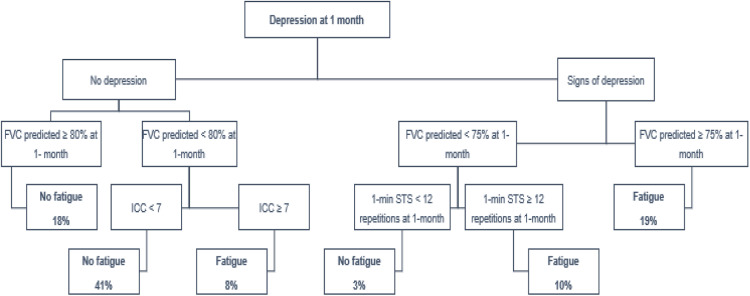
Predictors of persistent fatigue 12-months after hospital discharge. 1-min STS, 1-min Sit-to-Stand Test; CCI, Charlson Comorbidity Index; COVID-19, Coronavirus Disease of 2019; FVC, Forced Vital Capacity.

The decision tree model developed in our study demonstrated an accuracy of 79 %, with a sensitivity of 70 % and specificity of 87 % (95 % CI 0.7395–0.8347). Notably, the combination of depressive symptoms and predicted Forced Vital Capacity (FVC) values greater than 75 % after 30-days post-discharge proved to be significant, identifying these characteristics as potential factors associated with the persistence of long-term fatigue symptoms in 19 % of our sample (Fig. 3). Additionally, within the fatigue group, the decision tree indicated that the combination of depressive signs, decreased lung capacity with predicted FVC values <75 %, and impaired physical performance (defined as fewer than 12 repetitions per minute) accounted for 10 % of the sample (Fig. 3).

Decision tree model with the identification of factors associated with long-term fatigue symptom persistence.

## Discussion

In this study, the authors observed that approximately one-third of participants (36 %) continued to experience fatigue 12 months after hospital discharge due to COVID-19, with the highest prevalence reported in the first month post-discharge. Our findings are consistent with previous studies, such as one involving 88 participants, which reported a fatigue prevalence of 67 % at three months and 59 % at six months post-discharge.^27^ This outcome similarity may be attributed to the inclusion criteria in both studies, which focused on individuals requiring hospitalization. In contrast, a systematic review^28^ reported a lower pooled prevalence of fatigue at 28 % in the long term. This discrepancy may be explained by including studies encompassing patients with milder forms of the disease who were not hospitalized, potentially leading to reduced prevalence rates. Ultimately, our results highlight the need for further investigation into the mechanisms behind post-COVID fatigue and the factors that contribute to its persistence, such as the measurement of inflammatory biomarkers (interleukins, TNF-alpha, C-reactive protein, etc.), electromyographic and neurotransmitter conduction studies, particularly among patients with severe disease who require hospitalization. Understanding these mechanisms will be crucial for developing targeted interventions to alleviate fatigue in this population.

Our study also assessed perceived fatigue using a self-report questionnaire designed to evaluate participants' perceptions of the symptom and utilizing instruments that measured physical and mental exertion capacity.^29^ Notably, our findings indicated a trend toward reducing the prevalence of fatigue symptoms over the 12-month follow-up period. This decrease may be attributed to participants gradually returning to their activities, which likely contributed to a diminishing perception of fatigue despite objective signs of fatigue.

Our sample consisted mainly of individuals who were overweight. The low performance observed in 1-minute STS and pulmonary function may be attributed to the rapid and significant loss of lean mass experienced by participants who developed severe forms of the disease and required Intensive Care Unit (ICU) admission, as reported by Andrade-Junior et al.^30^ In their study, individuals exhibited an approximately 30 % reduction in the cross-sectional area of the rectus femoris muscle after just 10-days of hospitalization. The underlying mechanisms associated with this response to COVID-19 infection are not yet fully understood; however, they appear to mirror the muscle weakness commonly acquired during ICU stays.

Concerning physical capacity, our study found that the fatigue group performed significantly fewer repetitions compared to the non-fatigue group. Faria et al.^31^ reported similar findings, identifying fatigue in 48 % of 53 participants seven months after infection, among whom 24 % had been hospitalized. However, it is worth noting that participants in Faria et al.'s study appeared to be in better overall health; those with persistent post-COVID-19 symptoms were able to achieve an average of approximately 32 repetitions per minute (32.6 ± 7.0), while the fatigue group in our study averaged around 16 repetitions per minute (IQR 12‒21).

Our long-term fatigue prediction tree model did not detect the influence of gender, even considering that, after 12-months of follow-up, the most significant proportion of our sample was female. Previous studies indicate that the novel coronavirus more often infects men. Our findings align with the study by Huang et al.^32^ who observed that women appear more likely to experience symptoms of fatigue even after one year. There is evidence that X-chromosome-associated genes may be more vulnerable to viral infections and autoimmune diseases^33^ although this relationship is not fully understood.

The presence of multiple comorbidities one month after hospital discharge (ICC ≥ 7-points) appears to be associated with the long-term persistence of fatigue symptoms, as indicated by our predictive model. This finding aligns with a Canadian study,^27^ which reported similar results after three months, highlighting the number of comorbidities as a potential predictor for fatigue. However, the specific comorbidities most strongly related to long-term fatigue and their mechanisms of influence remain poorly understood.

Our analysis revealed that the fatigue group exhibited changes in lung capacity between the first and sixth months, with significant improvements only observed by the twelfth month. In contrast, individuals without fatigue had already achieved predicted Forced Vital Capacity (FVC) values above 80 % by the sixth month. A Brazilian study^34^ found that 76.5 % of participants recovering from COVID-19 exhibited one or more abnormalities on CT scans six months post-admission. The gradual recovery of lung capacity in the fatigue group at twelve months post-hospital discharge contrasts with findings from Cortes-Telles et al.^35^ and Sperling et al.,^36^ who noted no significant differences in pulmonary function tests between participants with and without fatigue, as predicted values had already surpassed 80 % after the three- and twelve-month follow-ups, respectively. It is well known that diaphragm excursion and chest wall mechanics are altered in individuals with obesity, contributing to reductions in FVC.^19^ This reduction has been associated with long COVID. Notably, our sample indicated that patients in the fatigue group had Body Mass Index (BMI) values exceeding 30 kg/m^2^.

Signs of anxiety and depression at 12-months post-COVID-19 infection were observed in our sample at high prevalence rates of 56 % and 66 %, respectively. These findings contrast with those reported by Han et al.,^28^ which indicated lower prevalence rates of 22 % and 23 % after 12-months. The authors believe this discrepancy can be attributed to the heterogeneity in the severity of participants' conditions across the included studies, as the limited understanding of COVID-19′s mechanisms and potential long-term sequelae ‒ particularly among severely affected individuals ‒ may lead to varied prognoses.

Several mechanisms have been proposed to explain the entry of the novel coronavirus into the Central Nervous System (CNS) and its potential relationship with the persistence of fatigue symptoms. One such mechanism involves glucose consumption by neuronal cells, which is fundamentally linked to neurotransmission processes and results in high energy demands.^37^ Additionally, studies have identified morphometric changes in the brains of COVID-19 patients, including signs of cerebellar hypermetabolism and frontal cortical hypometabolism.^37^ Another aspect that still needs to be explored is the involvement of the autonomic nervous system and its relationship with the symptoms of fatigue and depression, since the combination of an immunological imbalance possibly triggered by the activation of a chronic inflammatory cascade, can cause a pattern of brain fog, given the exacerbated activity of inflammatory cytokines that cross the blood-brain barrier and the process of hypoxia secondary to lung damage.^38^^,^^39^ New studies validating decision tree models in an external cohort are necessary to explore these underlying biological mechanisms in the development and intensity of fatigue.

The elevated prevalence of anxiety and depression in our sample, even one year after hospital discharge, may also reflect the impact of social distancing during the pandemic and the uncertainties it created about the future. In an underdeveloped country, concerns regarding the inability to return to work, economic instability, and fears of mortality due to the rampant spread of the virus, coupled with the loss of close contacts and initial difficulties in accessing vaccines, likely contributed to these negative psychological outcomes.

Although this study identifies associations between variables such as depression, fatigue, and lung function, it is an observational design, which does not allow for the establishment of causal relationships. Therefore, it is not possible to state, for example, whether depression contributes to increased fatigue or whether fatigue intensifies depressive symptoms. The findings should be interpreted as evidence of an association, useful for guiding future research with experimental designs or complementary longitudinal analyses that can explore potential causal relationships.

The chaotic circumstances surrounding the pandemic and the strain on healthcare systems posed significant challenges for research development, particularly in Brazil. Our study has limitations, and the interpretation of our findings should be approached with caution due to various restrictions encountered during the research process. The authors know that convenience samples can infer a possible selection bias, however, given the urgency for answers aimed at promoting adequate management of the disease and the need for inclusion restricted to participants who could attend the collection sites, such information raises the idea of the possibility of an underrepresentation of our sample, that is, whether it was mainly made up of those who were in better health on the days of the evaluations, by those who, even in a more serious condition, had the help of third parties to make their in-person participation possible or those who were more motivated to participate in the study. Additionally, it was not possible to establish comparisons with other populations, such as the inclusion of a control group containing patients who had COVID-19 but did not require hospitalization or healthy individuals, due to the social isolation measures recommended worldwide to prevent the spread and transmission of the new coronavírus.

The reduction in the number of participants is a common concern in longitudinal studies, as it represents an expected loss inherent to this type of study. However, the authors employed a statistical methodology that minimizes the impact of this limitation through the use of Generalized Linear Mixed Models (GLMM), which are robust in handling incomplete data resulting from attrition, under the assumption that the data are Missing At Random (MAR). These models allow the inclusion of all available observations at each time point, explicitly accounting for the correlation between repeated measures, which enhances the efficiency and reliability of the estimates, even in the presence of reduced sample sizes over time.

Finally, our study cannot include the assessment of fatigue symptoms through multidimensional instruments already validated for the Brazilian population, given that the existing instruments are not specific to the clinical condition of our participants and do not have cutoff points that would determine the absence of the symptom, mainly due to the lack of a control group containing healthy individuals for comparison. Furthermore, the authors chose to use the FACIT-F because it is an easy-to-apply instrument, covers aspects related to the physical, mental, functional, and social impact of fatigue, and is reliable for other clinical contexts. The values adopted in our study are based on references from international literature, which reinforces the need for future studies aimed at validating these parameters in national contexts, establishing cutoff points for various clinical conditions and different populations. The findings of this study provide an important foundation for the topic at hand; however, future studies with larger samples and independent cohorts could contribute to expanding and validating these findings.

In summary, our study offers valuable insights into the long-term persistence of fatigue and its association with physical performance, respiratory capacity, and psychological aspects in patients hospitalized for COVID-19. By conducting comprehensive screenings, this research underscores the need for a thorough assessment of the various systems involved in the development of fatigue, and the authors recognize that the present study did not focus on an in-depth analysis of fatigue subtypes or underlying pathophysiological mechanisms, since the main objective was to provide an initial characterization of the presence and intensity of this symptom in the studied sample. Therefore, the authors strongly encourage further investigations into this symptom's underlying mechanisms and impacts, ultimately guiding the formulation of health policies to enhance management and care for this population.

## Conclusion

Our study demonstrates that, while there is a downward trend in the prevalence of fatigue symptoms over time, a noteworthy 36.4 % of our sample continued to experience this symptom one year after hospital discharge. This finding is significant, particularly considering that many patients may not seek medical attention specifically for fatigue-related complaints. Additionally, the authors identified that symptoms of depression, in conjunction with impaired lung capacity one month post-discharge, may serve as potential predictors for the long-term persistence of post-COVID-19 fatigue. These results underscore the necessity for ongoing monitoring of fatigue symptoms through comprehensive multidisciplinary clinical evaluations, which is essential for ensuring appropriate therapeutic management for COVID-19 survivors. The authors recommend the implementation of early screening for depressive symptoms in clinical practice, using reliable and validated screening tools – including the HADS, as well as long-term monitoring of lung function, also with spirometry, within the first month post-discharge. Furthermore, the authors recommend including this population in personalized early rehabilitation programs that adopt a holistic approach to physical, pulmonary, and psychosocial aspects.

## Funding

This work was supported by the Fundação de Amparo à Pesquisa do Estado de São Paulo – FAPESP [process 19618-8/2018]; and the Conselho Nacional de Desenvolvimento – CNPq [process 402698/2020-7].

## CRediT authorship contribution statement

**Elizabeth Mendes da Silva:** Writing – review & editing, Writing – original draft, Methodology, Investigation, Supervision, Formal analysis, Data curation, Conceptualization. **Camila Machado de Campos:** Methodology, Investigation, Data curation. **Caroline Gil de Godoy:** Writing – original draft, Project administration, Methodology, Investigation, Supervision, Data curation. **Danielle Brancolini de Oliveira:** Methodology, Investigation, Data curation. **Angelica Castilho Alonso:** Visualization, Validation, Supervision, Investigation, Formal analysis. **Vanderlei Carneiro da Silva:** Validation, Supervision, Software, Formal analysis. **Ana Carolina Basso Schmitt:** Visualization, Validation, Supervision, Formal analysis. **Gabriela Sayuri Ochiai:** Investigation, Data curation. **Bianca de Oliveira Candido Viana:** Investigation, Data curation. **Juliana Magalhães da Silva:** Investigation. **Celso Ricardo Fernandes de Carvalho:** Validation, Supervision, Funding acquisition. **Carlos Roberto Ribeiro de Carvalho:** Resources, Investigation. **Julia Maria D'Andréa Greve:** Resources, Investigation. **José Eduardo Pompeu:** Writing – review & editing, Writing – original draft, Validation, Supervision, Resources, Project administration, Methodology, Formal analysis, Funding acquisition, Data curation, Conceptualization.

## Declaration of competing interest

The authors declare no conflicts of interest.

## Data Availability

Given the sensitive nature of the questions in this study, an anonymized version of the data may be provided to the corresponding author upon formal request, preserving the main study variables and the identification of participants and for exclusively academic and research purposes in accordance with ethical guidelines.
